# Haemotropic *Mycoplasma* species in cat blood samples by PCR

**DOI:** 10.17221/7/2025-VETMED

**Published:** 2025-08-30

**Authors:** Ozgul Gulaydin, Muazzez Yesilyurt, Gulsah Akgul, Ozlem Erdeger, Kerem Ercan

**Affiliations:** ^1^Department of Microbiology, Faculty of Veterinary Medicine, Siirt University, Siirt, Turkiye; ^2^Department of Internal Medicine, Faculty of Veterinary Medicine, Siirt University, Siirt, Turkiye

**Keywords:** *Candidatus* Mycoplasma haemominutum, feline, feline haemoplasma, molecular detection

## Abstract

Haemotropic mycoplasmas can cause severe anaemia in pets and also have zoonotic potential. The determination of these microorganisms from clinical samples is of critical importance. In this study, the presence of *Mycoplasma haemofelis* (Mhf), *Candidatus* Mycoplasma haemominutum (CMhm), and *Candidatus* Mycoplasma turicensis (CMt) in blood samples collected from 62 cats was investigated. Haemotropic mycoplasmas were identified by PCR amplifying the *16S rRNA* gene, and sequence analysis was applied for confirmation and differentiation of the species. Six (9.67%) blood samples were positive for haemotropic mycoplasmas. CMhm and CMt were identified in five (8.06%) samples and one (1.61%) sample, respectively. Mhf was not detected in the samples. Additionally, a co-infection was not observed in any of the cats. It was found that the PCR positivity was not related to the sex or clinical status of the cats. To our knowledge, this is the first report investigating haemotropic mycoplasmas in blood samples from cats in Siirt Province of Türkiye. It is suggested that the data obtained from this study will contribute to clinicians working on pet animal health in this province.

Haemotropic mycoplasmas, previously known as *Haemobartonella* spp. or *Eperythrozoon* spp., can cause haemolytic anaemia in a wide range of animal species. These microorganisms are Gram-negative, pleomorphic, and small erythrocytic parasites that cannot be cultured *in vitro* (Messick 2004). Following a 16S rRNA sequence analysis, *Haemobartonella* spp. and *Eperythrozoon* spp. were reclassified within the *Mycoplasmataceae* family and are now referred to as haemotropic mycoplasmas or haemoplasmas (Rikihisa et al. 1997; Messick 2004).

*Mycoplasma haemofelis* (Mhf) (previously known as the *Haemobartonella felis* Ohio strain), *Candidatus* Mycoplasma haemominutum (CMhm) (previously known as *Haemobartonella felis* California strain), and *Candidatus Mycoplasma turicensis* (CMt) are three haemotropic mycoplasmas that can cause feline infectious anaemia in wild and domestic cats (Willi et al. 2006a; Willi et al. 2006b). Mhf is the most pathogenic species among feline haemoplasmas. Severe anaemia is particularly observed in young cats infected with *M. haemofelis* (Tasker et al. 2003a; Tasker et al. 2009). In contrast, minimal clinical signs of acute disease are seen in cats experimentally infected with CMhm (Foley et al. 1998).

Haemotropic mycoplasmas can be transmitted among cats via the iatrogenic pathway, and arthropod vectors may play a role in their transmission (Taroura et al. 2005; Willi et al. 2006a; Willi et al. 2007). Since haemoplasma-like microorganisms have been identified in immunosuppressed humans, it is thought that haemotropic mycoplasmas may have zoonotic potential (Kallick et al. 1972; dos Santos et al. 2008).

Haemoplasmas can be diagnosed by staining blood smears with the Romanowsky method. They appear as coccoid forms on the surface of erythrocytes using this method. However, Romanowsky staining cannot differentiate between the species (Tasker et al. 2003b). Additionally, CMt cannot be detected in the blood samples of cats infected with this bacterium through microscopic examination. For these reasons, polymerase chain reaction (PCR) methods that amplify the *16S rRNA* gene have been developed (Willi et al. 2007). Although conventional PCR methods may fail to distinguish the species (Berent et al. 1998; Jensen et al. 2001; Criado-Fornelio et al. 2003), real-time PCR methods are more reliable (Willi et al. 2007).

The prevalence of haemotropic mycoplasmas has been investigated in various countries. Generally, CMhm is more common than the other species (Willi et al. 2006a; Willi et al. 2006b; Barker and Tasker 2013). In Türkiye, while Celik et al. (2021) reported that the prevalence of CMhm was higher than Mhf and CMt, Atalay et al. (2015) found that Mhf was more common. In this study, feline haemotropic mycoplasmas were investigated in blood samples of cats using PCR in Siirt Province, Türkiye.

## MATERIAL AND METHOD

### Sample collection

In this study, 62 blood samples were collected from cats brought to the Siirt University Animal Health Application and Research Centre between January 2022 and September 2022. Data such as the age, sex, the status of anaemia and icterus, and clinical diagnosis were recorded. The blood samples were collected from the antebrachial cephalic vein into sterile vacuum tubes with ethylenediaminetetraacetic acid (EDTA). The samples were sent to the microbiology laboratory and stored at –20 °C until DNA extraction.

### PCR analysis

Genomic DNA was isolated from the blood samples using a commercial kit (MG-KDNA-03; Hibrigen, Van, Türkiye). To detect CMhm, Mhf, and CMt, specific forward (5'-ACGAAAGTCTGATGGAGCAATA-3') and reverse (5'-ACGCCCAATAAATCCGRATAAT-3') primers were used (Jensen et al. 2001). These primers produced 193 bp amplicons for CMhm and 170 bp amplicons for Mhf and CMt.

To prepare a 25 μl of PCR mix, 12.5 μl of Mastermix (A.B.T^TM^ 2X PCR; Mastermix, Ankara, Türkiye), 1.5 μl of each primer (10 μM), 5 μl of genomic DNA, and 4.5 μl of PCR water were used.

The mix was held at 94 °C for 10 min for the initial denaturation. The amplification process was run with 35 cycles. The protocol consisted of denaturation at 94 °C for 1 min, annealing at 54 °C for 1 min, and extension at 72 °C for 1 minute. The final extension was applied at 72 °C for 10 minutes.

After amplification, the amplicons were electrophoresed on agarose gel at 80 V for 1.5 h and visualised on gel documentation system (Gen-Box imagER Fx; ERBiyotek, Ankara, Türkiye), compared with the 50 bp DNA ladder (DM012-R500; GeneDireX, Antalya, Türkiye).

### Sequence and phylogenetic analysis

A sequence analysis was applied to differentiate the species. Twenty-five μl of PCR products that had a specific band for the haemotropic mycoplasmas, along with each primer, were sent to the Laboratory of Hydra Biotechnology (Van, Türkiye) for a sequence analysis. The data obtained from the sequence analysis were compared with publicly available nucleotide sequences (NCBI BLAST) for confirmation.

The phylogenetic analysis was conducted with MEGA v11 software (Tamura et al. 2021). The evolutionary history of DNA sequences obtained from this study and the publicly available nucleotide sequences from GenBank were measured using the maximum likelihood method and the Tamura-Nei model (Tamura and Nei 1993). The phylogenetic tree was created via a bootstrap test (1 000 replicates) (Felsenstein 1985).

### Statistical analysis

The relationships between sex, anaemia status, icterus, and haemotropic mycoplasma positivity were analysed using Fisher’s Exact test method (Minitab Statistical Software v20 Statistical Package Programme, Pennsylvania, USA). A *P*-value of ≤0.05 was considered statistically significant.

### Ethical statement

This study was approved by the Local Ethics Committee for Animal Experiments at Siirt University with Decision No. 2022/01/05 on 28/02/2022.

## RESULTS AND DISCUSSION

In this study, blood samples from 62 cats brought to the Siirt University Animal Health Application and Research Centre with various complaints were utilised. The cats had an average age of 1.13 years (3 months–6 years). Thirty-three (53.23%) were female, and 29 (46.77%) were male. During the clinical examination, it was determined that 19 (30.64%) of the cats exhibited symptoms of anaemia, and 4 (6.45%) were icteric. Additionally, symptoms of enteritis, stomatitis, and pneumonia were detected in five (8.06%), three (4.83%), and one (1.61%) of the cats, respectively. One of the cats was found to be positive for haemotropic mycoplasma via microscopic examination. The remaining cats were brought in for routine check-ups or neutering operations.

**Figure 1:**
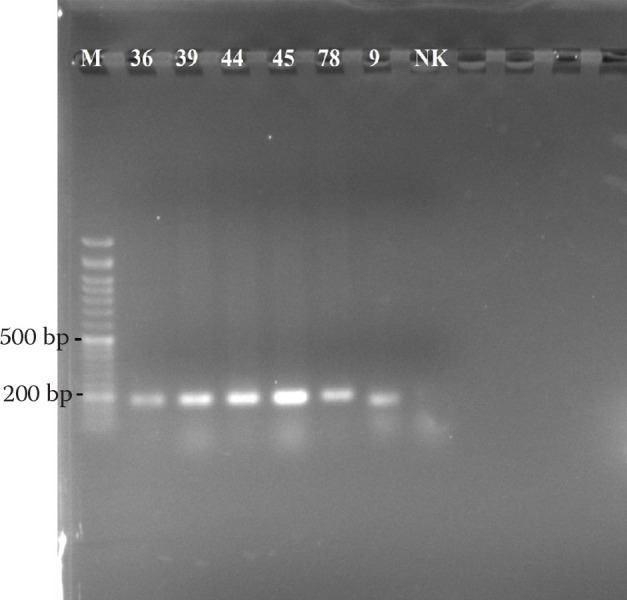
Image of amplicons on agarose gel (M: 50 bp DNA Ladder; 36, 39, 44, 78: *Candidatus Mycoplasma heamominutum* isolate (193 bp); 9: *Candidatus Mycoplasma turicensis* isolate (170 bp) NK = negative control without genomic DNA

**Figure 2:**
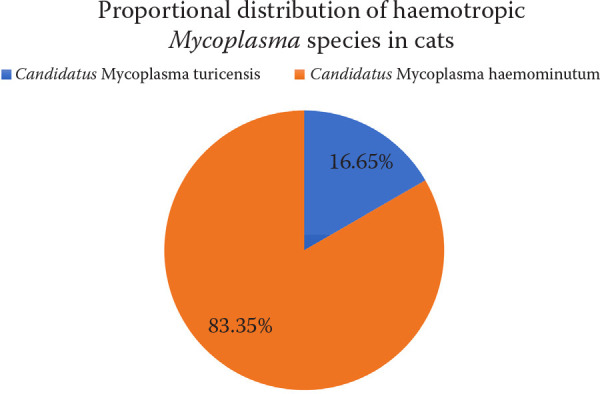
Pie chart showing the distribution of haemotropic *Mycoplasma* species (*Candidatus* Mycoplasma haemominutum and *Candidatus* Mycoplasma turicensis) detected by PCR in cat blood samples

Haemotropic mycoplasmas were detected in six (9.67%) of the blood samples through PCR. CMhm and CMt were identified in five (8.06%) and one (1.61%) of the samples, respectively (Figures 1 and 2). None of the samples tested positive for Mhf. A co-infection was not observed in any of the cats. Four (66.66%) haemotropic mycoplasma-positive cats were male. Furthermore, three (50%) of the positive cats had anaemia (Table 1). No statistical relationship was found between the sex, presence of anaemia or icterus, and haemotropic mycoplasma positivity (*P* > 0.05) (Table 2).

**Table 1:** Distribution of the age, sex, anaemia, and icterus status in haemoplasma-positive cats

No.	Age	Sex	Anaemia	Icterus	PCR
9	1 year	female	–	–	*Candidatus* Mycoplasma turicensis
36	6 years	male	+	–	*Candidatus* Mycoplasma haemominutum
39	4 months	male	–	–	*Candidatus* Mycoplasma haemominutum
44	6 years	female	+	–	*Candidatus* Mycoplasma haemominutum
45	1 year	male	–	–	*Candidatus* Mycoplasma haemominutum
78	3 years	male	+	–	*Candidatus* Mycoplasma haemominutum

**Table 2:** Association between the haemoplasma positivity and sex, status of anaemia, and icterus

Variable	Haemoplasma (+)	Haemoplasma (–)	*P-*value
Sex			
Female (*n* = 33)	2	31	0.304
Male (*n* = 29)	4	25	
Anaemia			
Positive (*n* = 19)	3	16	0.359
Negative (*n* = 43)	3	40	
Icterus			
Positive (*n* = 4)	0	4	1.000
Negative (*n* = 58)	0	58	

**Figure 3:**
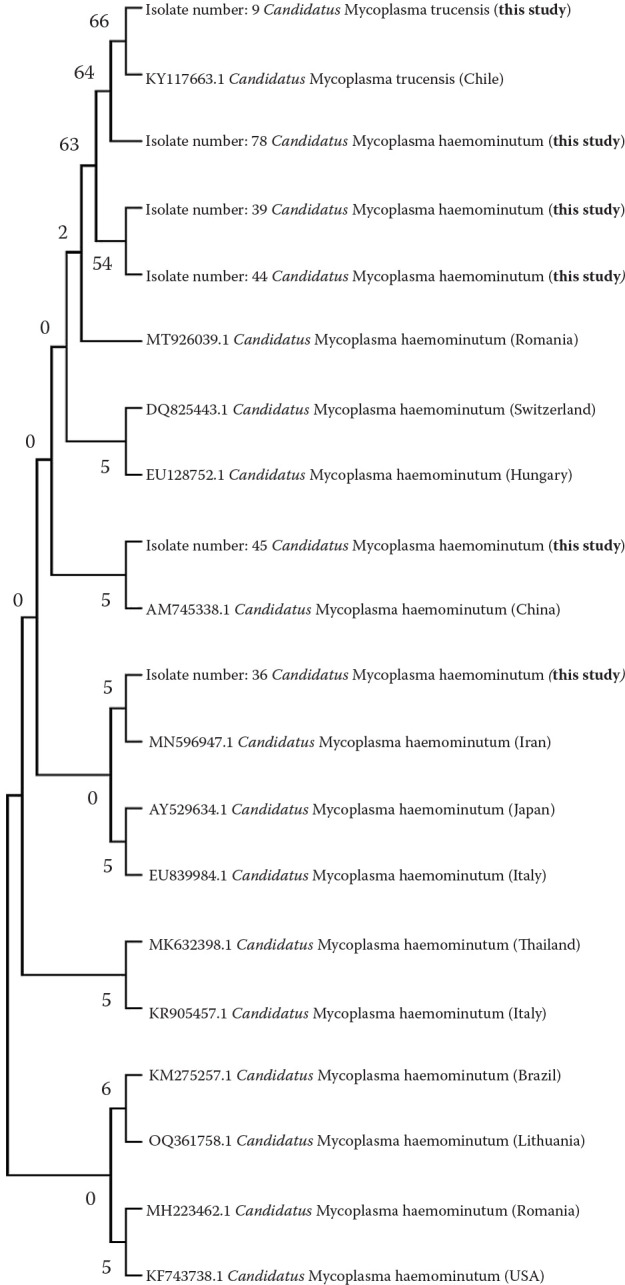
The bootstrap consensus tree inferred from 1 000 replicates shows that the evolutionary history of the taxa analysed Branches corresponding to partitions reproduced in less than 50% bootstrap replicates are collapsed. The percentage of replicate trees in which the associated taxa clustered together in the bootstrap test are shown next to the branches. Initial tree(s) for the heuristic search were obtained automatically by applying Neighbour-Join and BioNJ algorithms to a matrix of pairwise distances estimated using the Tamura-Nei model, and then selecting the topology with superior log likelihood value. This analysis involved 20 nucleotide sequences. There were a total of 248 positions in the final dataset

According to the Basic Local Alignment Search Tool (BLAST) analysis, the nucleotide sequence of CMt (Isolate No. 9) displayed 98% similarity to the sequence with Accession No. HE804777.1. Additionally, the nucleotide sequences of CMhm (Isolate Nos. 36, 39, 44, 45, 78) showed 97%, 100%, 100%, 99%, and 97% similarity to the sequences with Accession Nos. MN596947.1, MN240868.1, MN596947.1, MN240868.1, and MN543628.1, respectively. The phylogenetic tree illustrated the similarities between the sequences obtained from this study and the sequences isolated from cats in different countries (Figure 3).

In several studies conducted in various countries, haemoplasmas have been identified in samples taken from cats. In Greece, the prevalence of feline haemoplasma was found to be 20.6% (Maher et al. 2010). In Portugal, Duarte et al. (2015) and Martinez-Diaz et al. (2013) reported that haemoplasma was detected in 27.1% and 43.43% of cats, respectively. Diaz-Reganon et al. (2018) stated that this rate was 10.6% in Spain, and Sarvani et al. (2018) determined it as 17.2%. Furthermore, the prevalence of feline haemoplasmas was recorded as 17.7% in Japan (Inokuma et al. 2004), 13.8–25.8% in Brazil (Aquino et al. 2014; Makino et al. 2018), and 15.1% in Chile (Walker Vergara et al. 2016). Studies conducted in different regions of Italy revealed that 11.6–18.9% of cats were infected with haemotropic mycoplasmas (Gentilini et al. 2009; Ravagnan et al. 2017; Latrofa et al. 2020). In Türkiye, Ural et al. (2009) reported that the prevalence of feline haemoplasma was 18.9%. Similarly, Cetinkaya et al. (2016) determined that 19.3% of cats were infected with haemotropic mycoplasmas. Conversely, Celik et al. (2021) found that 8.54% of 246 cat blood samples were positive for haemoplasma in Istanbul province, Türkiye. In Kayseri province, feline haemoplasma was found in 9.52% of the blood samples (Atalay et al. 2015). Additionally, Ceylan et al. (2024) determined that the prevalence of haemotropic mycoplasma was 9.4% in blood samples of cats from Konya province. In line with advances in the field of molecular genetics, PCR techniques have increasingly been employed for the diagnosis of haemoplasma from blood samples collected from cats. Studies conducted in this context have reported that the specificity and sensitivity of the real-time PCR method are higher than those of conventional PCR (Sykes et al. 2007). In their respective studies, Duarte et al. (2015) and Latrofa et al. (2020) utilised the real-time PCR method for the diagnosis of haemoplasma. They reported higher rates of haemoplasma positivity compared to the present study. In the present study, feline haemoplasma was identified in 9.6% of the 62 blood samples from cats. This result aligns with the data reported by Atalay et al. (2015), Celik et al. (2021), and Ceylan et al. (2024), but is lower than the findings from other studies. It is thought that sample size, the clinical condition of the animals, and the diagnostic methods used in these studies may have influenced the results and cause variations in the prevalence of these microorganisms.

Generally, the rate of CMhm positivity is higher than Mhf in studies. It has been reported that between 7.6% and 47.0% of blood samples from cats tested positive for CMhm (Willi et al. 2006a; Fujihara et al. 2007; Peters et al. 2008; Cetinkaya et al. 2016; Rosenqvist et al. 2016; Latrofa et al. 2020; Celik et al. 2021; Demkin and Kazakov 2021). Similarly, in this study, the most prevalent feline haemoplasma was identified as CMhm (8.6%). CMt was found in 1.61% of the samples, while Mhf was not detected. Conversely, Kamrani et al. (2008) and Atalay et al. (2015) reported that the prevalence of Mhf was higher than CMhm. Although co-infections with varying rates were observed in other studies (Maher et al. 2010; Atalay et al. 2015; Celik et al. 2021; Demkin and Kazakov 2021), no cats in the present study were infected with two different haemoplasma species.

Haemotropic mycoplasmas can cause acute haemolytic anaemia in cats (Messick 2004). Although Mhf is reported to be the most widespread pathogenic species and can cause severe haemolytic anaemia in cats (Tasker et al. 2003a; Messick 2004), CMhm induces mild clinical signs or subclinical infections (Foley et al. 1998; Nibblett et al. 2010). Celik et al. (2021) found no association between the presence of anaemia or icterus and haemoplasma positivity. Conversely, another study revealed that anaemic cats infected with Mhf were more prevalent than apparently healthy cats (Ravagnan et al. 2017). Similar to Celik et al. (2021), the association between the presence of anaemia or icterus and haemoplasma positivity was not significant in this study. Haemotropic mycoplasmas have been reported to cause acute haemolytic anaemia in cats; however, there are also cases in which these agents do not lead to any clinical signs. Moreover, it has been emphasised that the acute or chronic stage of the infection plays a significant role in the manifestation of anaemic findings (Celik et al. 2021). In the present study, since the stage of infection was not monitored during sample collection and, unlike other studies (Ravagnan et al. 2017), the number of samples was limited, it is considered that no statistical association could be established between the haemoplasma positivity and the presence of anaemia.

In various studies, associations between feline immunodeficiency virus (FIV) and feline leukaemia virus (FeLV) and haemoplasma positivity in cats have been investigated, and a positive correlation between these parameters has been identified (Inokuma et al. 2004; Gentilini et al. 2009; Duarte et al. 2015; Martinez-Diaz et al. 2013; Ravagnan et al. 2017). However, in this study, clinical or laboratory diagnoses of viral diseases were not performed on the cats. This is considered one of the limitations of the present study, and it is suggested that future research should focus on determining the relationship between the haemoplasma positivity and diseases that cause immunosuppression in cats.

In this study, feline haemoplasma was identified in 9.67% of the blood samples taken from cats. No statistical relationship was found between the sex, presence of anaemia or icterus, and haemoplasma positivity. Based on the data reported in previous studies, future research conducted in the Siirt province of Türkiye should aim to investigate haemoplasma positivity in a larger feline population and explore potential predisposing factors associated with infection. It is thought that the data obtained from this study will contribute to the knowledge of pet clinicians in this province and the wider literature.
